# Persistent mycotic superficial femoral artery pseudoaneurysm after endovascular treatment: a case report

**DOI:** 10.1590/1677-5449.200095

**Published:** 2021-09-14

**Authors:** Ruth Fuente, Francisco J. Medina, Natalia Moradillo, Ignacio Agúndez, Mónica Herrero, Victoria Santaolalla

**Affiliations:** 1 Burgos University Hospital, Vascular Surgery Department, Burgos, Spain.

**Keywords:** mycotic pseudoaneurysm, endovascular treatment, stent thrombosis, Escherichia coli, arterial ligation, superficial femoral artery, pseudoaneurisma micótico, tratamento endovascular, trombose de stent, Escherichia coli, ligadura arterial, artéria femoral superficial

## Abstract

Mycotic pseudoaneurysms of the superficial femoral artery (SFA) are rare and are usually secondary to colonization of an atherosclerotic plaque during an episode of bacteremia. We describe the case of a 68 year-old diabetic male who presented to the Emergency Department with pyrexia and a painful expanding mass in the left thigh. He had a history of diarrhea and had been treated 16 days earlier for an SFA pseudoaneurysm that had been excluded with a covered stent with no adjunctive antibiotic therapy. Angio CT showed an abscess surrounding femoral vessels and stent thrombosis. Under general anesthesia, we performed extensive debridement, removal of the endovascular material, SFA ligation, and empirical antibiotic therapy. Blood and tissue cultures were positive for *Escherichia coli*. At the 3-months follow up visit, the patient reported he had no claudication. In selected patients, mycotic pseudoaneurysms can be treated by SFA ligation.

## Resumo

Pseudoaneurismas micóticos da artéria femoral superficial (AFS) são raros, e geralmente são secundários à colonização de uma placa aterosclerótica durante bacteremia. Relatamos o caso de um paciente masculino diabético de 68 anos que chegou ao Serviço de Emergência com pirexia e massa expansiva dolorosa na coxa esquerda. Apresentava histórico de diarreia e havia sido tratado 16 dias antes para pseudoaneurisma da AFS, que foi excluído com stent coberto e sem antibioticoterapia adjuvante. A angiotomografia computadorizada demonstrou um abscesso ao redor dos vasos femorais e trombose do stent. Sob anestesia geral, realizamos desbridamento extenso, remoção do material endovascular, ligadura de AFS e antibioticoterapia empírica. Culturas de sangue e tecidos foram positivas para *Escherichia coli*. Na consulta de seguimento aos 3 meses, o paciente negou claudicação. Em pacientes selecionados, pseudoaneurismas micóticos podem ser tratados com ligadura de AFS.

## INTRODUCTION

Mycotic aneurysms account for 2.5% to 3% of all aneurysms.^1^^,^ 2 Those involving the femoral artery are even rarer (0.8% of mycotic aneurysms).^3^ Clinical presentation is nonspecific and a high degree of clinical suspicion is required to arrive at the correct diagnosis. Misdiagnosis can lead to inappropriate treatment. As rheumatic heart valve disease declines and the population ages, Gram-negative bacteria such as Salmonella sp and *Escherichia coli* account for an increasing proportion of pathogens associated with arterial infection.^2^ We present a case of femoral artery pseudoaneurysm caused by *E. Coli* that was initially treated with endovascular techniques in which the clinical course was complicated by abscess formation.

In our institution clinical cases do not need to be approved by the Institutional Research Committee, only prospective studies where there is an intervention. Informed consent was obtained for studies tha involved humans and the manuscript is in accordance with the Helsinki Declaration and with local ethical guidelines.

## CLINICAL CASE

**Figure 1:**
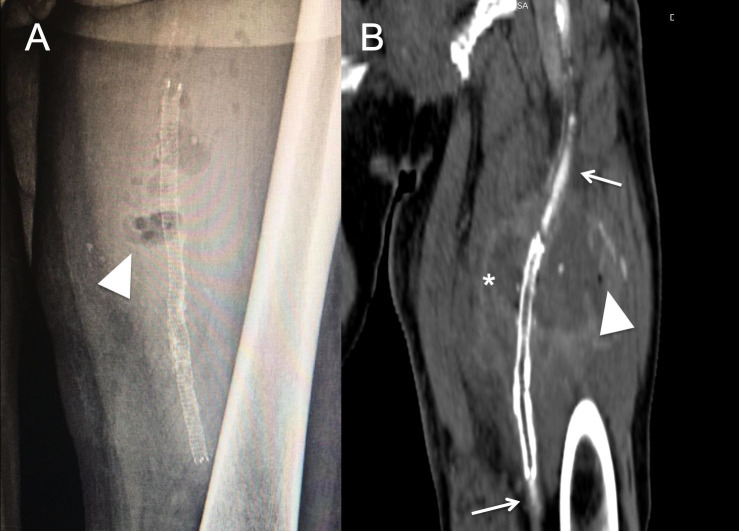
(A) Plain X-ray, stent (arrow) surrounded by air bubbles (arrowhead); (B) Angio CT reconstruction, thrombosed stent (arrow), and large pseudoaneurysm (asterisk).

**Figure 2:**
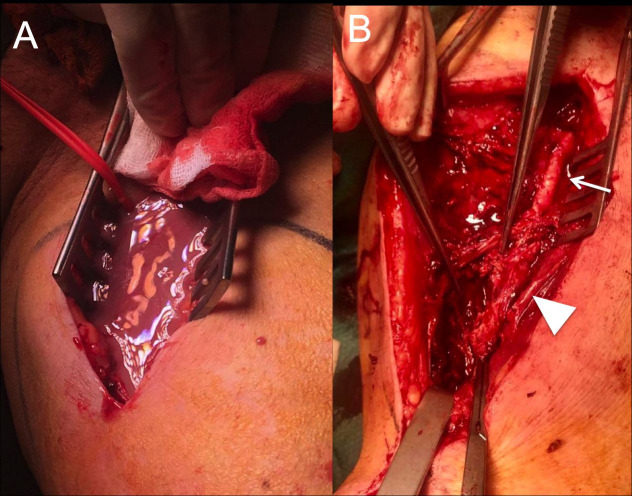
Intraoperative findings. (A) pseudoaneurysm abscessification; (B) complete destruction of superficial femoral artery (arrow).

A 68-year-old male was admitted to the Emergency Department with pyrexia and a painful expanding mass in the left thigh. The patient was an ex-smoker and had a history of type II diabetes and hypertension. He had been admitted to another hospital for the same symptoms 16 days earlier. An Angio CT had shown a left superficial femoral artery (SFA) pseudoaneurysm. This was treated by exclusion with a covered self-expanding stent graft (6x 100mm) (Viabahn ®, W. L. Gore & Associates, Flagstaff, Ariz) via ipsilateral femoral artery cutdown. The patient mentioned progressive swelling and pain in the left thigh, despite the intervention. He denied claudication prior to or after the intervention. He also reported an episode of self-limiting diarrhea one month before. At his arrival to our centre, the patient was hemodynamically stable and febrile (38.1 °C). On physical examination, all pulses were present in the right lower limb, but only the femoral pulse was palpable in the left lower limb. The left thigh was swollen and erythematous, with raised local temperature. The femoral surgical incision also showed signs of inflammation. The laboratory test was significant for elevated white cell count (15.2 × 10^9^/L) and elevated C reactive protein (117 mg/L). Blood cultures were ordered. Duplex ultrasound showed a spherical 10 cm hypoechoic structure surrounding the left femoral vessels and stent graft thrombosis. Doppler waveforms in the left popliteal and distal arteries were monophasic and the ABI index in that limb was 0.45. A plain radiography showed air bubbles in the soft tissues around the stent (Figure 1A). Angio CT confirmed the diagnosis of superficial femoral artery pseudoaneurysm abscess and stent thrombosis (Figure 1B). Emergency surgery was proposed. As the patient did not mention ischemic symptoms, the surgical plan included abscess debridement, endovascular material resection, and superficial femoral artery ligation. The procedure was performed under general anesthesia. Proximal vascular control was obtained through the previous incision. The SFA was accessed in the middle of the thigh. Once the superficial fascia was open, 700 mL of purulent exudate drained from the incision (Figure 2A). The middle portion of the SFA was severely damaged (Figure 2B). The covered stent was then removed, the SFA was ligated, and extensive debridement was performed. Pus, the stent, and a segment of the artery were sent for cultures. Empirical antibiotic therapy was started with piperacilline-tazobactam. Cultures of the abscess, stent graft, and blood samples were positive for *Escherichia coli* extended spectrum betalactamase responding to fluoroquinolones. The patient did not develop ischemic symptoms in the left lower limb, despite a postoperative AB index of 0.45. During the postoperative period, the wound developed dehiscence, which was treated with negative pressure dressings. The patient was discharged on the 13th postoperative day. At three-month follow-up, the patient was doing well, the wound had healed, the AB index of the affected limb was 0.5, and he did not have left lower limb claudication.

## DISCUSSION

Osler was the first to describe mycotic aneurysms, in 1885.^4^ In his series, the aneurysm was caused by septic emboli originating in valve vegetations of patients with bacterial endocarditis. The term “mycotic” refers to the mushroom shape of the aneurysm and has no relation to the etiology of the infection. Nowadays, the term arterial infection is preferred to avoid confusion with aneurysms caused by fungi. Four mechanism of arterial infection have been described: 1. Infection caused by septic cardiac emboli as described by Osler; 2. Infection of a pre-existing aneurysm (caused by bacteremia or contiguous spread); 3. Posttraumatic infected pseudoaneurysms in drug abusers; and 4. Microbial arteritis (caused by bacteremia or contiguous spread).^5^ In the postantibiotic era, posttraumatic infection related to drug abuse and microbial arteritis are the most prevalent causes of arterial infection.

Healthy arteries are resistant to bacterial infection because the endothelial cells act as a barrier to microorganism invasion. Presence of an aneurysm or more frequently an arteriosclerotic plaque constitutes a homeostatic fissure and a gateway to bacterial seeding.^2^ Once that happens, collagenase produced by pathogens but also elastolytic activity produced by host leucocytes destroy the arterial wall with subsequent pseudoaneurysm formation.^6^^,^ 7 Arterial infections more commonly affect patients who are immunocompromised with chronic diseases, such as diabetes mellitus, chronic renal insufficiency, cancer, or rheumatoid arthritis, but prior arterial catheterization, operation at a remote site, or chronic use of corticosteroid agents can also increase propensity.^8^^,^ 9 Our patient was a diabetic patient and although he did not report claudication, he had cardiovascular risk factors that might have led to SFA plaque formation.

Over the last 50 years, changes in the evolution of the etiology of arterial infections have generated modifications in their bacteriology. Before 1960, mycotic aneurysms resulted from heart valve disease, with the arterial infection caused by Gram-positive bacteria such as *Streptococcus pyogenes, Streptococcus pneumoniae*, and *Staphylococcus aureus*.^5^ Since then, an increase of Gram-negative organisms has been observed, especially Salmonella species, but also Escherichia coli.^2^ Although the pathophysiology remains unclear, it seems that after ingestion some patients will develop salmonella gastroenteritis, and some of those will develop bacteremia with extraintestinal seeding and infection. Higgins et al.^10^ have proved the affinity of Salmonella for the arterial wall in the elderly and in patients with diabetes. Although rare, *E. coli* has been reported infecting arteries at different locations such as supraaortic trunks and the ascending and abdominal aorta.^11^^-^ 14 In our case, the presentation of the infection in the post-operative period established a differential diagnosis of arteritis or secondary graft infection; in the latter, culture results would have included Gram positive bacteria. Furthermore, the history of previous self-limiting diarrhea may explain the positive cultures for *E. Coli*, making a diagnosis of primary infection more plausible. A high degree of suspicion is needed to arrive at a diagnosis of infected arteritis, since in the first stages of the disease the symptoms are usually very unspecific and vary depending on the artery involved. General symptoms include fever, hyporexia, nocturnal diaphoresis, and even a constitutional syndrome.^15^ As far as femoral arteries are concerned, Patra et al.^2^ analyzed a series of patients in which they found an 83% incidence of pain and fever. In this context, the pain is presumably due to rapid aneurysm expansion. Rupture was the presenting symptom in 25% of the patients. Laboratory findings may include leukocytosis and augmented acute phase reactants that are sensitive but not specific to the diagnosis.^15^^,^ 16 Blood cultures are also helpful prior to the operation, but the diagnosis is not ruled out if they come out negative.^16^ Contrast-enhanced tomography is usually the preferred imaging exam in the emergency setting.

Antibiotic therapy is crucial in treatment of arterial infection. Once the diagnosis has been made, broad spectrum antibiotic therapy should be started, and then switched to targeted antibiotics once the results of the cultures are available.^16^ It is recommended that treatment should be continued for at least 6 weeks. Although they can be curative in aortic infection if diagnosed in the early stages,^17^ antibiotics are usually an adjunctive but necessary complement to surgery. In our case, the patient had been previously misdiagnosed and the endovascular treatment on its own was ineffective. The standard treatment for mycotic aneurysms has for years consisted of surgical resection and extensive debridement with or without revascularisation.^2^^,^ 15 In-situ or extraanatomical bypass can be used. If in-situ revascularization is performed, graft conduits include autologous veins (saphenous, femoral). Cryopreserved allograft is also an option if available.^2^^,^ 15 In case of extra- anatomical bypass such as trans-obturator or ilio-femoral, rifampicin-soaked or silver-impregnated grafts are preferred. In our case, since the patient presented an asymptomatic thrombosis of the stent, we decided on primary arterial ligation. Recently, endovascular treatment has emerged as an alternative to open surgery in the urgent setting. This approach can be curative, if associated with aggressive long-term antibiotic therapy or a primary “damage control” phase, to prevent arterial rupture followed by intravenous antibiotic treatment and open surgical repair once the infection is controlled.^18^ In a recent study, Xu et al.^19^ presented good results with use of covered stents for treatment of superficial femoral artery pseudoaneurysms in 29 drug abuser with covered stent grafts. At 9 months there was 100% patency and no recurrent symptoms.

This case illustrates the importance of a correct diagnosis in a patient with fever and a painful expanding process in the thigh. Endovascular treatment with no adjunctive antibiotic therapy should not be performed in this situation. Open surgery with direct arterial ligation without reconstruction, extensive debridement, and graft removal can be safely performed in selected patients.
